# Total metagenomes outperform viromes in recovering viral diversity from sulfuric soils

**DOI:** 10.1093/ismeco/ycae017

**Published:** 2024-01-27

**Authors:** Li Bi, Ji-Zheng He, Hang-Wei Hu

**Affiliations:** School of Agriculture, Food and Ecosystem Sciences, Faculty of Science, The University of Melbourne, Parkville, Victoria 3010, Australia; School of Agriculture, Food and Ecosystem Sciences, Faculty of Science, The University of Melbourne, Parkville, Victoria 3010, Australia; School of Agriculture, Food and Ecosystem Sciences, Faculty of Science, The University of Melbourne, Parkville, Victoria 3010, Australia

**Keywords:** soil viruses, total metagenomes, viromes, viral diversity, acid sulfate soils

## Abstract

Recent metagenomic advancements have offered unprecedented insights into soil viral ecology. However, it remains a challenge to select the suitable metagenomic method for investigating soil viruses under different environmental conditions. Here, we assessed the performance of viral size-fraction metagenomes (viromes) and total metagenomes in capturing viral diversity from hypersulfidic soils with neutral pH and sulfuric soils with pH <3.3. Viromes effectively enhanced the sequencing coverage of viral genomes in both soil types. Viomes of hypersulfidic soils outperformed total metagenomes by recovering a significantly higher number of viral operational taxonomic units (vOTUs). However, total metagenomes of sulfuric soils recovered ~4.5 times more vOTUs than viromes on average. Altogether, our findings suggest that the choice between viromes and total metagenomes for studying soil viruses should be carefully considered based on the specific environmental conditions.

## Main text

Viruses are highly abundant on Earth and contribute significantly to biogeochemical cycles. Total metagenomes, extracting all microbial DNA, have been used to study the soil virosphere [1, 2]. However, the vast diversity of soil microbiota and “relic” DNA poses obstacles to the reconstruction of viral genomes using total metagenomes [3, 4]. To address these limitations, researchers have turned to soil viral size-fraction metagenomes (viromes) for reducing cellular contamination and enhancing viral particle concentration [5]. Several studies demonstrated that viromes outperformed total metagenomes in uncovering rare virosphere in agriculture and peatland soils [6, 7]. However, viromes also have drawbacks, including low DNA yields, extraction of only free viral particles, and labour-intensive procedures [5]. The broader applicability of viromes remains relatively unexplored due to significant variations in soil biophysiochemical properties across different types [8]. Therefore, it becomes imperative to evaluate the effectiveness of viromes versus total metagenomes across diverse soil types for accurately understanding soil viral dynamics.

Here, we collected 36 samples from Adelaide, South Australia, including two types of acid sulfate soils (18 hypersulfidic soil samples with neutral pH from a current mangrove swamp on the Garden Island and 18 sulfuric soil samples with pH <3.3 from adjacent disturbed areas). Since 1935, the disturbed area has experienced reclamation for agriculture and industry, leading to the oxidation of sulfidic materials and generation of substantial acid and sulfuric soils [9, 10]. We constructed both virome and total metagenome libraries to retrieve the viral diversity, following established protocols [7, 11] (see Supplementary materials).

**Figure 1:**
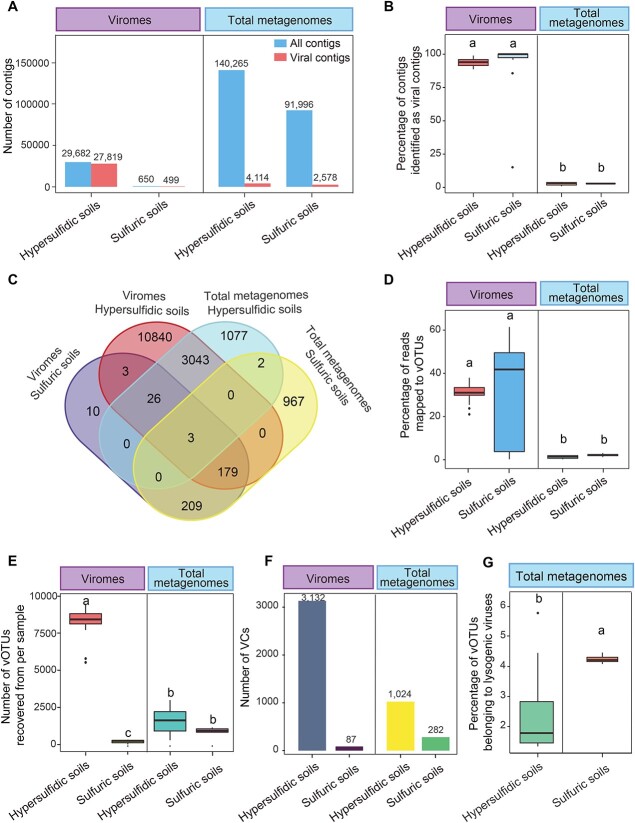
Comparisons of the performance of viromes and total metagenomes in recovering viral diversity from hypersulfidic and sulfuric soils. (A) The number of all contigs assembled and viral contigs identified from viromes and total metagenomes of hypersulfidic and sulfuric soils. (B) The average percentage of contigs belonging to viral genomes, determined by per virome and total metagenome from both soil types. (C) Venn diagram showing the shared and unique vOTUs among the two soil types with the two metagenomic methods. (D) The percentage of reads mapped to vOTUs, assessed by each virome and total metagenome from hypersulfidic and sulfuric soils. (E) The number of vOTUs recovered per virome and total metagenome based on the reads mapping to all vOTUs. (F) The size of viral clusters from viromes or total metagenomes in hypersulfidic and sulfuric soils. (G) Percentage of putative lysogenic viruses in total metagenomes of the two soil types. Different letters above the boxes show the significant difference determined by the Kruskal–Wallis nonparametric test.

The virome and total metagenome sequencing yielded approximately 850 and 870 Gb of raw data, respectively. For viromes, 29 682 contigs (> 10 kb) were assembled from hypersulfidic soils and 650 from sulfuric soils (Fig. 1A). About 93.7% and 76.8% of contigs were identified as viral contigs in hypersulfidic and sulfuric soils, respectively (Fig. 1B). In contrast, for total metagenomes, 232 261 contigs were assembled (Fig. 1A), while the proportion of viral contigs was considerably lower, at 2.9% for hypersulfidic soils and 2.8% for sulfuric soils (Fig. 1B). Consistent with previous findings [6, 7], viromes showed a notable 20 to 30-fold increase in the recovery percentage of viral contigs compared to total metagenomes. All viral contigs were clustered into 16 359 viral operational taxonomic units (vOTUs) (Fig. 1C). On average, 30.7% and 45.0% of virome reads from hypersulfidic and sulfuric soils, respectively, were mapped to vOTUs, higher than that of total metagenomes (~1%–2%) (Figure 1D). These results highlight the effectiveness of viromes in enriching viral particles compared to total metagenomes.

Viromes of hypersulfidic soils revealed the highest number of vOTUs (14094), followed by total metagenomes of hypersulfidic soils (4151) and sulfuric soils (1360), and viromes of sulfuric soils (430) (Fig. 1C). The accumulation curves for vOTUs reached saturation (Fig. S1), suggesting that our sampling effort was adequate to capture the richness of vOTUs. In hypersulfidic soils, each virome yielded 5.1 times more vOTUs compared to total metagenomes. Conversely, in sulfuric soils, the viral richness per total metagenome exceeded viromes by 4.5 times (Fig. 1E). This aligns with observations from human gut viral metadata, where total metagenomes identified more viral contigs than viromes [12]. Furthermore, vOTUs were grouped into viral clusters (VCs) using vConTACT2 (Figs 1F and S2) [13]. The number of VCs clustered from the viromes of hypersulfidic was higher than that from total metagenomes, while sulfuric soils showed an opposite trend (Fig. 1F). These results suggest that viromes outperform total metagenomes in capturing higher viral diversity from hypersulfidic soils, whereas sulfuric soils, considered as extreme environments, showed an opposite pattern.

The better performance of total metagenomes over viromes in sulfuric soils may be mainly attributed to the prolonged extreme acidity of the environment, which may directly impact the persistence of most free viral particles [14, 15]. Additionally, previous studies showed that heavy metals can significantly elevate lysogenic virus abundance [16], and in some extreme environments, one or more viruses were present in nearly every cell [17]. In sulfuric soils, harsh conditions may induce the shift from lytic to lysogenic viral strategies for survival adaption. This is partially supported by the significantly higher proportion of lysogenic vOTUs in the total metagenome of sulfuric soils compared to hypersulfidic soils (Fig. 1G). Those factors collectively render viromes less effective in capturing prevalent viral signals within this environment (Table S1). Moreover, technical factors can also influence the performance of different metagenomes in capturing the diversity of soil viruses. For instance, the steps involved in virome DNA extraction and library preparation may lead to the reduction of viral diversity in sulfuric soils. Additionally, the lower biological complexity in extreme environments, such as sulfuric soils, may contribute to an enhanced efficiency of vOTU recovery from total metagenomes [5, 18].

Viromes have been widely recognized as a powerful tool for studying soil viral ecology. Our results support viromes as a promising approach for studying viruses in natural soil systems. However, in extreme environments such as sulfuric soils, total metagenomes can be the initial consideration due to their efficiency and lower labour requirements in capturing a comprehensive viral profile. Moreover, future studies should encompass a broad array of soil types to refine the application of metagenomic methods in characterizing the soil virosphere.

## Supplementary Material

ISME_Communication_submission_SM_20240118_ycae017_new

## Data Availability

Raw sequencing data from this study were submitted to the NCBI under the project PRJNA1016489. All viral genomes can be accessed at FigShare (https://doi.org/10.6084/m9.figshare.25016585.v1).

## References

[1] Ji M , Fan X, Cornell CR et al. Tundra soil viruses mediate responses of microbial communities to climate warming. MBi*o* 2023;14:e03009–22. 10.1128/mbio.03009-22 36786571 PMC10127799

[2] Emerson JB , Roux S, Brum JR et al. Host-linked soil viral ecology along a permafrost thaw gradient. Nat Microbio*l* 2018;3:870–80. 10.1038/s41564-018-0190-y 30013236 PMC6786970

[3] Roux S , Emerson JB. Diversity in the soil virosphere: to infinity and beyond? Trends Microbio*l* 2022;30:1025–35. 10.1016/j.tim.2022.05.003 35644779

[4] Bi L , Yu D-T, Han L-L et al. Unravelling the ecological complexity of soil viromes: challenges and opportunities. Sci Total Enviro*n* 2022;812:152217. 10.1016/j.scitotenv.2021.152217 34890674

[5] Trubl G , Hyman P, Roux S et al. Coming-of-age characterization of soil viruses: a User’s guide to virus isolation, detection within metagenomes, and Viromics. Soil System*s* 2020;4:23. 10.3390/soilsystems4020023

[6] Ter Horst AM , Santos-Medellín C, Sorensen JW et al. Minnesota peat viromes reveal terrestrial and aquatic niche partitioning for local and global viral populations. Microbiom*e* 2021;9:1–18 34836550 10.1186/s40168-021-01156-0 PMC8626947

[7] Santos-Medellin C , Zinke LA, Ter Horst AM et al. Viromes outperform total metagenomes in revealing the spatiotemporal patterns of agricultural soil viral communities. ISME *J* 2021;15:1956–70. 10.1038/s41396-021-00897-y 33612831 PMC8245658

[8] Fierer N . Embracing the unknown: disentangling the complexities of the soil microbiome. Nat Rev Microbio*l* 2017;15:579–90. 10.1038/nrmicro.2017.87 28824177

[9] Fitzpatrick R . Demands on Soil Classification and Soil Survey Strategies: Special-Purpose Soil Classification Systems for Local Practical Us*e*. Dordrecht: Springer, 2013

[10] Poch R , Thomas BP, Fitzpatrick R et al. Micromorphological evidence for mineral weathering pathways in a coastal acid sulfate soil sequence with Mediterranean-type climate, South Australia. Soil Re*s* 2009;47:403–22. 10.1071/SR07015

[11] Bi L , Yu D-T, Du S et al. Diversity and potential biogeochemical impacts of viruses in bulk and rhizosphere soils. Environ Microbio*l* 2021;23:588–99. 10.1111/1462-2920.15010 32249528

[12] Gregory AC , Zablocki O, Zayed AA et al. The gut virome database reveals age-dependent patterns of virome diversity in the human gut. Cell Host Microb*e* 2020;28:724–740.e8 e8. 10.1016/j.chom.2020.08.003 32841606 PMC7443397

[13] Bin Jang H , Bolduc B, Zablocki O et al. Taxonomic assignment of uncultivated prokaryotic virus genomes is enabled by gene-sharing networks. Nat Biotechno*l* 2019;37:632–9. 10.1038/s41587-019-0100-8 31061483

[14] Bagdasaryan G . Survival of viruses of the enterovirus group (poliomyelitis, ECHO, Coxsackie) in soil and on vegetables. J Hyg Epidemiol Microbiol Immuno*l* 1964;8:497–505 14238948

[15] Thomas ED , Reichelderfer CF, Heimpel AM. The effect of soil pH on the persistence of cabbage looper nuclear polyhedrosis virus in soil. J Invertebr Patho*l* 1973;21:21–5. 10.1016/0022-2011(73)90108-0

[16] Huang D , Yu P, Ye M et al. Enhanced mutualistic symbiosis between soil phages and bacteria with elevated chromium-induced environmental stress. Microbiom*e* 2021;9:1–15. 10.1186/s40168-021-01074-1 34183048 PMC8240259

[17] Munson-McGee JH , Peng S, Dewerff S et al. A virus or more in (nearly) every cell: ubiquitous networks of virus–host interactions in extreme environments. ISME *J* 2018;12:1706–14. 10.1038/s41396-018-0071-7 29467398 PMC6018696

[18] Shu W-S , Huang L-N. Microbial diversity in extreme environments. Nat Rev Microbio*l* 2022;20:219–35. 10.1038/s41579-021-00648-y 34754082

