# Supplementary material for: Total metagenomes outperform viromes in recovering viral diversity from sulfuric soils
Source: ISME Commun. 2024 Jan 27;4(1):ycae017. doi: 10.1093/ismeco/ycae017 (PMC10936692; doi:10.1093/ismeco/ycae017)
Supplement: ISME_Communication_submission_SM_20240118_ycae017_new [file isme_communication_submission_sm_20240118_ycae017_new.docx]

***Supplementary materials***

**Total metagenomes outperform viromes in recovering viral diversity from sulfuric soils**

Li Bi ^a^, Ji-Zheng He ^a^, Hang-Wei Hu ^a,*^

*^a^* School of Agriculture, Food and Ecosystem Sciences, Faculty of Science, The University of Melbourne, Parkville, Victoria 3010, Australia

*Corresponding author:

Hang-Wei Hu, Email: hang-wei.hu@unimelb.edu.au

**Materials and methods**

**Soil sampling**

We collected a total of 36 soil samples from Barker Inlet estuary close to Adelaide (South Australia) (34°48′22″-34°49′47″ S, 138°31′42″-138°32′39″ E). This region has a Mediterranean climate with a mean annual temperature and precipitation of 16.2 °C and 517 mm, respectively. Eighteen samples were collected from three distinct sites in the mangrove swamp on the Garden Island. At each site, a total of six samples were collected from six different soil layers ranging from 20 to 110 cm in depth. Another 18 samples were collected from three sites of adjacent drained mangrove swamp, with sampling depths spanning from 5 to 100 cm. This disturbed area has been reclaimed from the intertidal and supratidal environments of Barker Inlet since the 1930s by construction of a series of bund walls that prevented tidal inundation for agriculture and industry. The loss of tidal inundation has resulted in lower water table, enabling oxygen to diffuse into sulfidic materials, leading to the generation of substantial sulfuric acidity and the formation of sulfuric materials (pH < 4) [1, 2].

Approximately 1.5 kg of each soil sample was stored in a clean zip-lock bag with minimal air to reduce the impacts of oxygen. Soil samples were transported to the laboratory within two days. Each soil sample was evenly mixed and divided into two parts. The first part was stored at 4 °C for the extraction of viral particles, and another part was stored at -80 °C for total DNA extraction.

**Soil pH determination and acid sulfate soil classification**

To identify the classification of acid sulfate soil materials, we immediately tested the soil pH when they soil arrived as field soil pH, and the soil pH after an eight-week period of aerobic incubation as oxidized soil pH according to the Australian Soil Classification [3]. Soil pH was assessed with a soil-to-water ratio of 1:1 (w/w) using a pH meter (HI5221, Hanna, Australia). The field soil pH of the 18 samples from the mangrove swamp was about 6.5~7.2. Among them, 15 samples with the oxidized pH below 3 were classified as hypersulfidic materials. The remaining three samples from the top layer with the oxidized pH beyond 4 were classified as hyposulfidic materials. In accordance with the Australian Soil Classification [3], the soil profile was classified as a Sapric Histic-Hypersulfidic, Intertidal Hydrosol and as Subaquatic/Tidalic, Histic Gleysol (Hypersulfidic, Arenic) according to the WRB identification keys [4]. The soil was classified as a hypersulfidic soil in accordance with the Australian acid sulfate soil classification (Fitzpatrick, 2013). The field soil pH of another 18 samples collected from the drained area at Gillman was below 3.3 and jarosite was visually observed at the collection site. Those samples were classified as sulfuric soils with sulfuric materials in accordance with the Australian acid sulfate soil classification [2].

**DNA extraction and preparation of metagenome sequencing**

Viromes were generated with some modifications from previous studies [5, 6]. Briefly, 40 g of soil was used for viral particle extraction with the modified PPBS buffer (10% phosphate-buffered saline, 150 mM MgSO_4_, 1% K Citrate and 1% bovine serum albumin). The suspension was filleted using 0.45 and 0.22 μm Millex filters (Millipore, Tullagreen, Ireland), and concentrated using 30 kDa Amicon Ultra centrifugal filter units (Millipore, Tullagreen, Ireland). The concentration was treated with DNase I (10 units/100 μL; Thermo Fisher Scientific, Vilnius, Lithuania) for 40 mins at 37 °C to remove extracellular DNA contamination, and subsequently incubated with EDTA at 65 °C for 10 mins. The cellular DNA was checked by running 16S rRNA gene PCR amplification [7] and agarose gel electrophoresis revealed no bands. The viral DNA was extracted by the Qiagen AllPrep PowerViral DNA/RNA extraction kit (Qiagen, Hilden, Germany), followed by being fragmented to approximately 350 bp using the Covaris S2 system (Covaris, Woburn, MA, USA). The fragmented DNA was purified using the Zymo Research DNA Clean and Concentrator kit (Zymo Research, Orange, CA, USA). Virome libraries were generated with the xGen ssDNA & Low-Input DNA Library Prep Kit (Integrated DNA Technologies, Coralville, IA, USA) following the manufacturer’s recommendations. The quantification of viral libraries was carried out using a 1× High Sensitivity DNA assay with the Invitrogen Qubit 2 Fluorometer (Invitrogen, Carlsbad, CA, USA).﻿ Three replicate negative controls were included throughout the virome DNA extraction and library preparation process. The concentration of libraries in negative controls was below 0.5 ng µl^-1^, which did not meet the sequencing threshold and thus were excluded from downstream analysis.

For total metagenome, soil DNA was extracted from 0.25 g of soil with the DNeasy PowerSoil Pro Kit (Qiagen, Hilden, Germany) and the sequencing libraries were generated with Illumina DNA Prep (Illumina, San Diego, CA, USA) at the Australian Genome Research Facility (AGRF) in Melbourne, Australia, following the manufacturer’s recommendations. DNA extraction for six samples of sulfuric soils was unsuccessful despite our efforts to explore alternative DNA extraction methods [8], likely due to the exceptionally low input of genetic material and significant contamination, such as heavy metals. Both virome and total metagenome were sequenced using the NovaSeq S4 platform with 2 × 150 bp read length at the AGRF.

**Bioinformatics analyses and statistical analyses**

Viromes yielded an average of 78,102,742 reads and total metagenome yielded an average of 96,166,950 reads for each sample. Fastp (v0.23.2) was used for raw data processing with default parameters [9], and contigs were assembled from individual libraries using Megahit (v1.1.2) with default parameters [10]. Viral contigs (> 10 kb) were identified with VIBRANT v1.2.1 (with the virome mode for contigs generated from viromes) and VirSorter2 v2.2.4 [11, 12]. Viral contigs identified by either of the two methods were used for downstream analyses. All viral contigs were grouped into species-level viral operational taxonomic units (vOTUs) using cd-hit with 95% identity and 80% coverage [13]. Subsequently, the quality of all vOTUs was assessed using CheckV [14]. All qualified reads were mapped to vOTUs using CoverM 0.6.1 (<https://github.com/wwood/CoverM>) with the method of Read Per Kilobase per Million mapped reads (RPKM), average nucleotide identity ≥ 90%, and coverage ≥ 75%. A vOTU was considered to be present in the virome or metagenome of a soil sample, if it had an RPKM > 0. If the RPKM was 0, the vOTU was deemed absent in the virome or total metagenome of the soil sample.

The putative lysogenic viral genomes were identified using VIBRANT v1.2.1. Open reading frames (ORFs) of all vOTUs were predicted by Prodigal 2.6.3 with metagenome mode [15]. The produced amino acid file was used to build a gene-sharing network using vConTACT2 v0.9.19 on the CyVerse (<https://de.cyverse.org/de>) with NCBI Bacterial and Archaeal Viral RefSeq (v85) as the reference database and Diamond used for calculating protein-protein similarity [16], generating the approximate genus-level viral clusters (VCs). The sharing network of reference sequences and vOTUs was visualized in Cytoscape v3.6.0 [17].

For the assessment of viral diversity retrieved from both viromes and total metagenomes in hypersulfidic and sulfuric soils, data normality was assessed using the Shapiro-Wilk test, and statistical testing was conducted using the Kruskal-Wallis nonparametric test. Figures were visualized using ImageGP72 (<http://www.ehbio.com/ImageGP/>) [18]. Accumulation curves of vOTUs were calculated based on the presence of the vOTU in viromes or total metagenomes of each soil sample and generated using the “speccacum” function in R v4.2.1 [19].

References

1. Poch R, Thomas BP, Fitzpatrick R, Merry R. Micromorphological evidence for mineral weathering pathways in a coastal acid sulfate soil sequence with Mediterranean-type climate, South Australia. *Soil Res* 2009;**47**(4):403-22.

2. Fitzpatrick R. Demands on soil classification and soil survey strategies: special-purpose soil classification systems for local practical use. Springer, Dordrecht; 2013.

3. Isbell R, Terrain NCoSa. The Australian soil classification, Third Edition. Melbourne, Australia: CSIRO publishing; 2021.

4. WRB IWG: World reference base for soil resources 2014, update 2015. In: International soil classification system for naming soils and creating legends for soil maps World Soil Resources Reports No 106 FAO, Rome. 2016.

5. Bi L, Yu D-T, Du S, Zhang L-M, Zhang L-Y, Wu C-F *et al*. Diversity and potential biogeochemical impacts of viruses in bulk and rhizosphere soils. *Environ Microbiol* 2021;**23**(2):588-99.

6. Santos-Medellin C, Zinke LA, Ter Horst AM, Gelardi DL, Parikh SJ, Emerson JB. Viromes outperform total metagenomes in revealing the spatiotemporal patterns of agricultural soil viral communities. *ISME J* 2021;**15**(7):1956-70.

7. Bates ST, Berg-Lyons D, Caporaso JG, Walters WA, Knight R, Fierer N. Examining the global distribution of dominant archaeal populations in soil. *ISME J* 2011;**5**(5):908-17.

8. Fang Y, Xu M, Chen X, Sun G, Guo J, Wu W *et al*. Modified pretreatment method for total microbial DNA extraction from contaminated river sediment. *Front Env Sci Eng* 2015;**9**:444-52.

9. Chen SF, Zhou YQ, Chen YR, Gu J. fastp: an ultra-fast all-in-one FASTQ preprocessor. *Bioinformatics* 2018;**34**(17):884-90.

10. Li D, Liu C-M, Luo R, Sadakane K, Lam T-W. MEGAHIT: an ultra-fast single-node solution for large and complex metagenomics assembly via succinct de Bruijn graph. *Bioinformatics* 2015;**31**(10):1674-6.

11. Guo J, Bolduc B, Zayed AA, Varsani A, Dominguez-Huerta G, Delmont TO *et al*. VirSorter2: a multi-classifier, expert-guided approach to detect diverse DNA and RNA viruses. *Microbiome* 2021;**9**(37).

12. Kieft K, Zhou Z, Anantharaman K. VIBRANT: automated recovery, annotation and curation of microbial viruses, and evaluation of viral community function from genomic sequences. *Microbiome* 2020;**8**:1-23.

13. Fu L, Niu B, Zhu Z, Wu S, Li W. CD-HIT: accelerated for clustering the next-generation sequencing data. *Bioinformatics* 2012;**28**(23):3150-2.

14. Nayfach S, Camargo AP, Schulz F, Eloe-Fadrosh E, Roux S, Kyrpides NC. CheckV assesses the quality and completeness of metagenome-assembled viral genomes. *Nat Biotechnol* 2020;**39**:578-85.

15. Hyatt D, Chen G-L, LoCascio PF, Land ML, Larimer FW, Hauser LJ. Prodigal: prokaryotic gene recognition and translation initiation site identification. *BMC bioinformatics* 2010;**11**(1):119.

16. Bin Jang H, Bolduc B, Zablocki O, Kuhn JH, Roux S, Adriaenssens EM *et al*. Taxonomic assignment of uncultivated prokaryotic virus genomes is enabled by gene-sharing networks. *Nat Biotechnol* 2019;**37**(6):632-9.

17. Shannon P, Markiel A, Ozier O, Baliga NS, Wang JT, Ramage D *et al*. Cytoscape: A software environment for integrated models of biomolecular interaction networks. *Genome Research* 2003;**13**(11):2498-504.

18. Chen T, Liu YX, Huang L. ImageGP: An easy‐to‐use data visualization web server for scientific researchers. *Imeta* 2022;**1**(1):e5.

19. Oksanen J. Vegan: community ecology package. [*http://CRAN*](http://CRAN) *R-project org/package= vegan* 2010.

**Figure legends**

**Figure S1** Accumulation curves for vOTUs in viromes (A, all vOTUs from both hypesulfidic and sulfuric soils; B, vOTUs from hypesulfidic soils; C, vOTUs from sulfuric soils) and total metagenomes (D, all vOTUs from both hypesulfidic and sulfuric soils; E, vOTUs from hypesulfidic soils; F, vOTUs from sulfuric soils).


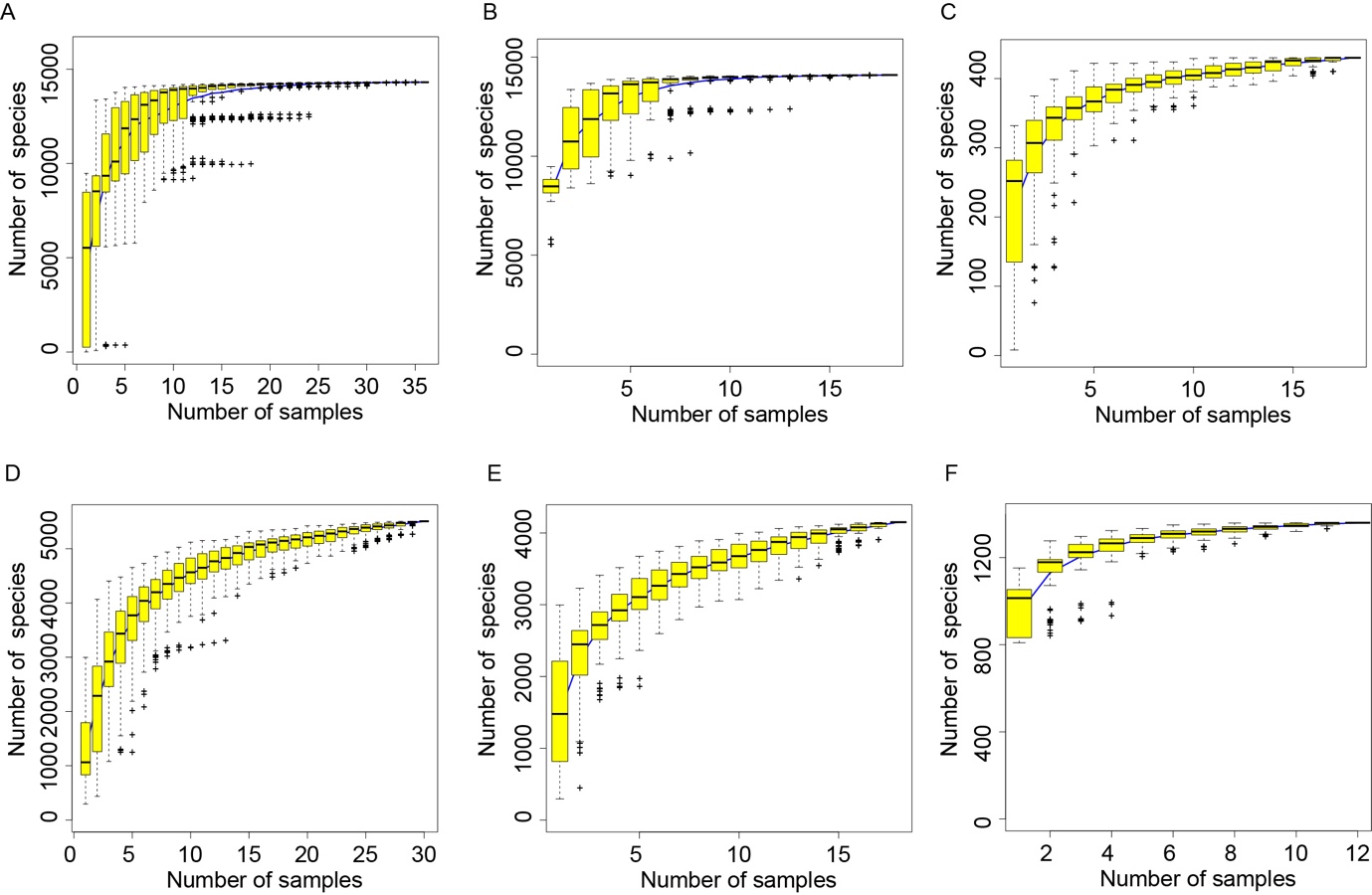


**Figure S2** A gene-sharing network for vOTUs unique to viromes of hypesulfidic soils (light bule), total metagenome (TM) of hypesulfidic soils (green), unique to viromes and TM of hypesulfidic soil (blue), unique to viromes of sulfuric soils (orange), unique to TM of sulfuric soils (purple), unique to viromes and TM of sulfuric soils (light purple), the rest of vOTUs shared in viromes and TM of hypesulfidic and sulfuric soils (yellow), and the NCBI RefSeq database (gray). Nodes represent viral genomes and edges connect viral genomes that shared protein contents predicted from vConTACT2. If a set of nodes have considerably higher edge weights than the rest of the network they are linked to, these are grouped together to form viral clusters (VCs).


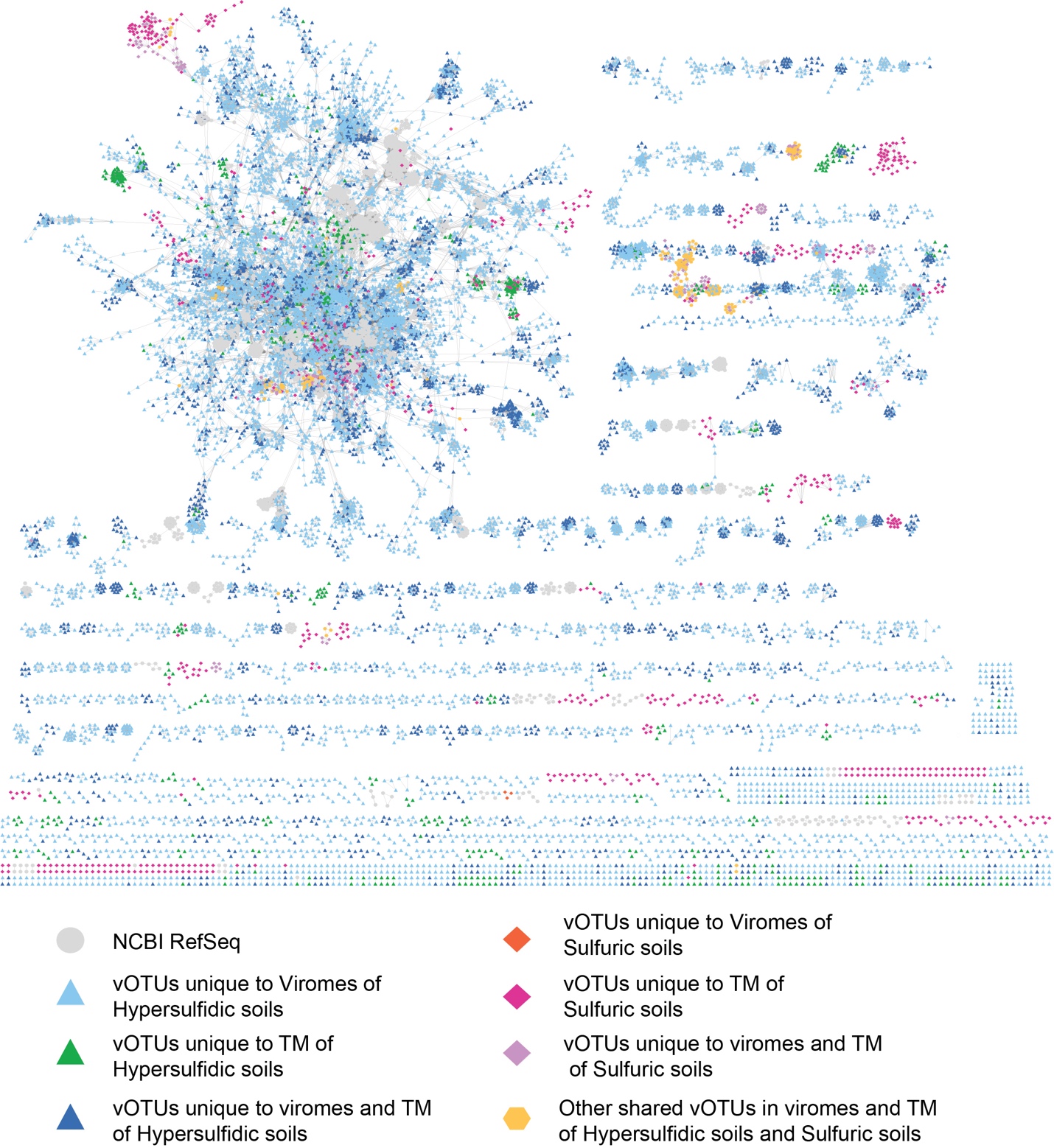


**Table S1** The library yields of viromes from hypersulfidic soils and sulfuric soils and negative controls.

| Sample labels | Soil types | Concentrations (ng µl^-1^) |
| --- | --- | --- |
| 1 | Hypersulfidic soils | 41.20 |
| 2 | Hypersulfidic soils | 34.60 |
| 3 | Hypersulfidic soils | 27.20 |
| 4 | Hypersulfidic soils | 30.60 |
| 5 | Hypersulfidic soils | 38.80 |
| 6 | Hypersulfidic soils | 21.20 |
| 7 | Hypersulfidic soils | 40.20 |
| 8 | Hypersulfidic soils | 43.80 |
| 9 | Hypersulfidic soils | 40.00 |
| 10 | Hypersulfidic soils | 38.20 |
| 11 | Hypersulfidic soils | 37.60 |
| 12 | Hypersulfidic soils | 39.00 |
| 13 | Hypersulfidic soils | 34.60 |
| 14 | Hypersulfidic soils | 46.00 |
| 15 | Hypersulfidic soils | 24.20 |
| 16 | Hypersulfidic soils | 25.40 |
| 17 | Hypersulfidic soils | 30.20 |
| 18 | Hypersulfidic soils | 30.80 |
| 19 | Sulfuric soils | 2.28 |
| 20 | Sulfuric soils | 4.88 |
| 21 | Sulfuric soils | 4.65 |
| 22 | Sulfuric soils | 5.66 |
| 23 | Sulfuric soils | 2.40 |
| 24 | Sulfuric soils | 3.36 |
| 25 | Sulfuric soils | 8.98 |
| 26 | Sulfuric soils | 14.20 |
| 27 | Sulfuric soils | 10.20 |
| 28 | Sulfuric soils | 5.26 |
| 29 | Sulfuric soils | 3.92 |
| 30 | Sulfuric soils | 4.92 |
| 31 | Sulfuric soils | 12.80 |
| 32 | Sulfuric soils | 11.90 |
| 33 | Sulfuric soils | 14.10 |
| 34 | Sulfuric soils | 8.14 |
| 35 | Sulfuric soils | 12.70 |
| 36 | Sulfuric soils | 7.80 |
| 37 | Negative control | < 0.5 |
| 38 | Negative control | < 0.5 |
| 39 | Negative control | < 0.5 |
